# The prognostic value of the stress hyperglycemia ratio for all-cause and cardiovascular mortality in patients with diabetes or prediabetes: insights from NHANES 2005–2018

**DOI:** 10.1186/s12933-024-02172-8

**Published:** 2024-02-28

**Authors:** Lei Ding, Hongda Zhang, Cong Dai, Aikai Zhang, Fengyuan Yu, Lijie Mi, Yingjie Qi, Min Tang

**Affiliations:** 1 Department of Cardiology, State Key Laboratory of Cardiovascular Disease, Cardiovascular Institute, Fuwai Hospital, National Center for Cardiovascular Diseases, Chinese Academy of Medical Sciences, Peking Union Medical College, No.167 Beilishi Rd, Xicheng District, Beijing, 100037 China; 2 Department of Health Policy and Management, School of Public Health, Peking University Health Science Center, Beijing, 100191 China

**Keywords:** Stress hyperglycemia ratio (SHR), All-cause mortality, Cardiovascular mortality, Diabetes, Prediabetes

## Abstract

**Background:**

The Stress hyperglycemia ratio (SHR) is a novel marker reflecting the true acute hyperglycemia status and is associated with clinical adverse events. The relationship between SHR and mortality in patients with diabetes or prediabetes is still unclear. This study aimed to investigate the predictive value of the SHR for all-cause and cardiovascular mortality in patients with diabetes or prediabetes.

**Methods:**

This study included 11,160 patients diagnosed with diabetes or prediabetes from the National Health and Nutrition Examination Survey (2005–2018). The study endpoints were all-cause and cardiovascular mortality, and morality data were extracted from the National Death Index (NDI) up to December 31, 2019. Patients were divided into SHR quartiles. Cox proportion hazards regression was applied to determine the prognostic value of SHR. Model 1 was not adjusted for any covariates. Model 2 was adjusted for age, sex, and race. Model 3 was adjusted for age, sex, race, BMI, smoking status, alcohol use, hypertension, CHD, CKD, anemia, and TG.

**Results:**

During a mean follow-up of 84.9 months, a total of 1538 all-cause deaths and 410 cardiovascular deaths were recorded. Kaplan-Meier survival analysis showed the lowest all-cause mortality incidence was in quartile 3 (*P* < 0.001). Multivariate Cox regression analyses indicated that, compared to the 1st quartile, the 4th quartile was associated with higher all-cause mortality (model 1: HR = 0.89, 95% CI 0.74–10.7, *P* = 0.226; model 2: HR = 1.24, 95% CI 1.03-1.49, *P* = 0.026; model 3: HR = 1.30, 95% CI 1.08–1.57, *P* = 0.006). The 3rd quartile was associated with lower cardiovascular mortality than quartile 1 (model 1: HR = 0.47, 95% CI 0.32–0.69, *P* < 0.001; model 2: HR = 0.66, 95% CI 0.45–0.96, *P* = 0.032; model 3: HR = 0.68, 95% CI 0.46–0.99, *P* = 0.049). There was a U-shaped association between SHR and all-cause mortality and an L-shaped association between SHR and cardiovascular mortality, with inflection points of SHR for poor prognosis of 0.87 and 0.93, respectively.

**Conclusion:**

SHR is related to all-cause and cardiovascular mortality in patients with diabetes or prediabetes. SHR may have predictive value in those patients.

## Background

The epidemic of diabetes mellitus (DM) and its complications pose a major global health threat. As estimated by the International Diabetes Federation (IDF), 1 in 10 adults aged ≥ 20–79 years had DM globally in 2021, and this estimate is expected to increase to 783.2 million by 2045 [1]. DM often has an onset years before it is diagnosed, especially if this DM includes prediabetes. The United States has the third highest prevalence of DM, and half of adults aged over 65 years have prediabetes [1]. The complications of DM are traditionally divided into microvascular and macrovascular complications, including coronary heart disease, peripheral vascular disease, cerebrovascular disease, renal failure, and diabetic retinopathy [2–4]. In addition, patients with DM have higher risk of all-cause and cardiovascular mortality [5]. Therefore, early identification of high-risk patients and the discovery of more risk factors are essential for improving patient prognosis.

Recently, numerous studies have shown that hyperglycemia in hospitalized patients is associated with increased morbidity and mortality in patients with myocardial infarction, heart failure, chronic obstructive pulmonary disease, cerebrovascular disease and critical illness [6–10]. However, the blood glucose level at admission cannot reveal the chronic glucose level. Glycosylated hemoglobin type A1c (HbA1c) is a well-established marker of glycemia over the previous 8 to 12 weeks, as it can reflect the estimated average glucose concentration. Therefore, a novel marker named the stress hyperglycemia ratio (SHR) was devised. It is calculated from the admission glucose and HbA1c levels [11]. A higher SHR was confirmed to be a risk factor for cardiovascular disease [8, 12, 13] in patients with or without DM. Recently, a meta-analysis of 26 cohort studies revealed that acute myocardial infarction patients with higher SHR had a significantly higher risk of major adverse cardiovascular and cerebrovascular events (MACCE), long-term all-cause mortality, and in-hospital all-cause mortality than patients with lower SHR. Subgroup analysis yielded the same conclusion regardless of diabetes status [14]. However, the relationship between the SHR and mortality risk has not been clearly investigated in diabetic or pre-diabetic patients.

Thus, this study aimed to investigate the relationship between SHR and all-cause and cardiovascular mortality in a large, nationally representative population of diabetes or prediabetes patients in the United States (US).

## Methods

### Study population

**Fig. 1:**
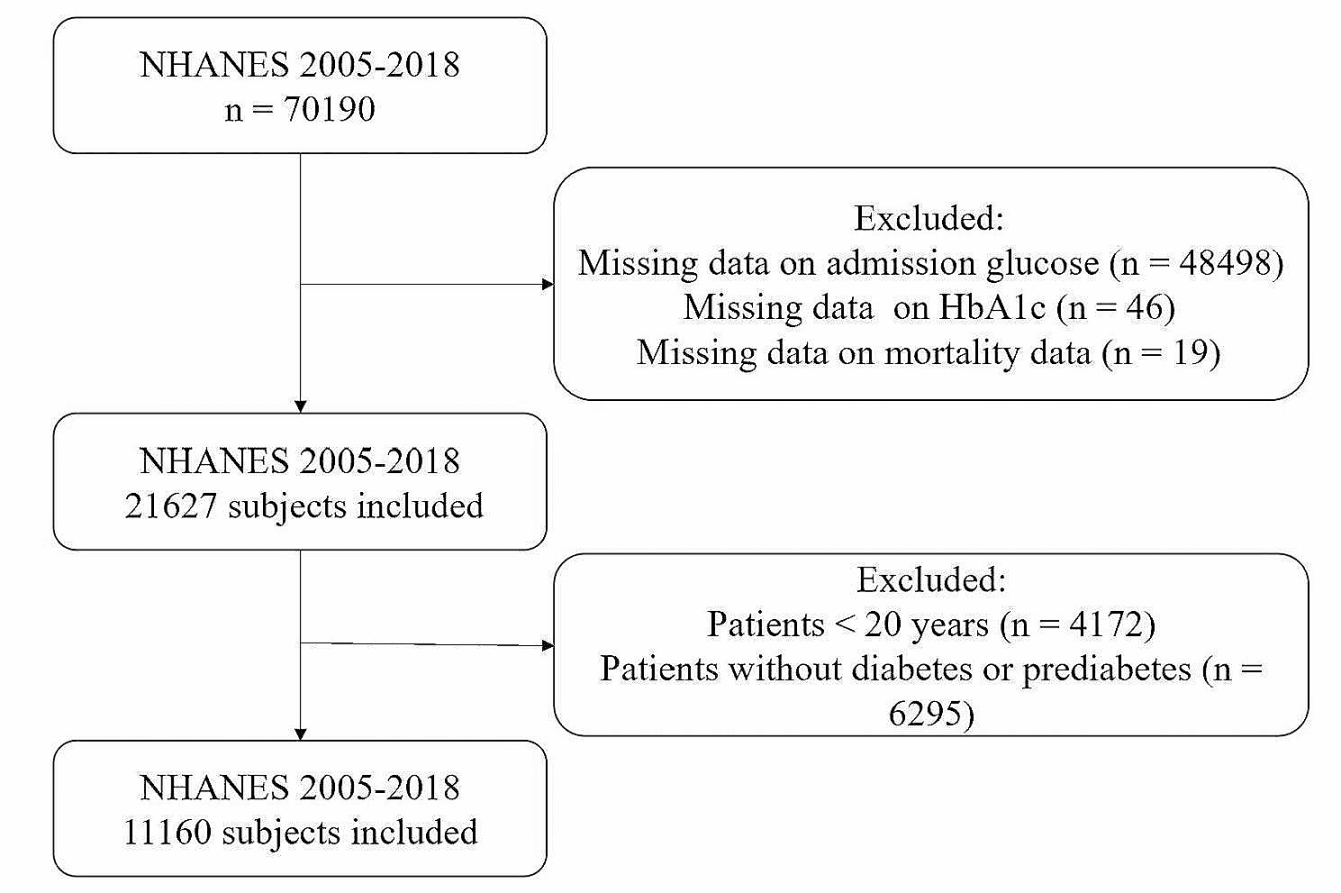
Flowchart of the sample selection from NHANES 2005–2018

The data used in this study were extracted from the National Health and Nutrition Examination Survey (NHANES), which is a program managed by the Centers for Disease Control and Prevention (CDC) and the National Centers for Health Statistics (NCHS) in the US. This program follows the STROBE guidelines for reporting observational studies. The protocols of NHANES were approved by the Research Ethics Review Board of the NCHS, and informed written consent was obtained from all of the participants involved in the study. We downloaded the data from the NHANES website for 2005–2018 (https://www.cdc.gov/nchs/nhanes/index.htm), covering seven survey cycles. The data analysed in the current study included demographic data, examination data, laboratory data, and questionnaire data. According to the ADA’s diabetes diagnostic criteria [15], diabetes was defined as having any of the following conditions: (a) HbA1c concentration ≥ 6.5% or fasting plasma glucose (FPG) level ≥ 126 mg/dL; (b) a response ‘yes’ to the question: ‘Doctor told you have diabetes?’ or ‘Taking insulin now?’. Prediabetes status was defined as having any of the following: (a) HbA1c concentration between 5.7% and 6.4% or FPG level between 100 mg/dL and 125 mg/dL; (b) response ‘yes’ to the question: ‘Doctor told you have prediabetes?’. A total of 70,190 participants were screened in the NHANES cohort between 2005 and 2018. After excluding those with missing data on admission glucose (n = 48498), HbA1c (n = 46), mortality data (n = 19), patients younger than 20 years old (n = 4172), or without diabetes or prediabetes (n = 6295), we included 11,160 eligible patients with diabetes or prediabetes in the final analysis (Fig. 1).

### Study variables

Demographic, examination, laboratory and questionnaire data were downloaded from the NHANES website. BMI was calculated as weight in kilograms divided by the square of height in meters and was grouped into underweight (< 18.5), normal (18.5 to < 25), overweight (25 to < 30), and obese (≥ 30). Race was categorized as Mexican American, other Hispanic, non-Hispanic white, non-Hispanic black or other races. Smoking status was classified as never-smoker, former smoker or current smoker. Individuals were considered nondrinkers, 1 to < 5 drinks/month, 5 to < 10 drinks/month, or 10 + drinks/month. Hypertension status was determined from a self-reported medical history of high blood pressure, antihypertensive medicine, or non-same-day randomized records of 3 times of systolic blood pressure ≥ 140 mmHg or diastolic blood pressure ≥ 90 mmHg. Coronary heart disease (CHD) was diagnosed as self-reported CHD, angina pectoris or myocardial infarction. Chronic kidney disease was determined by a response of ‘yes’ to the question “Ever told you had weak/failing kidneys?”. Anemia was defined as hemoglobin < 120 g/L in males and < 110 g/L in females. Laboratory data assessed in this study including FPG, HbA1c, total cholesterol, low-density lipoprotein cholesterol (LDL-C), high-density lipoprotein cholesterol (HDL-C), triglycerides (TG), hemoglobin, were extracted from the NHANES website.

### Calculation of SHR

SHR was calculated by the formula [FPG (mmol/L)]/[1.59 * HbA1c (%)-2.59] [11]. FPG was performed by the Fairview Medical Center Laboratory at the University of Minnesota. HbA1c measurements were done by the Diabetes Laboratory at the University of Minnesota using a Tosoh A1c 2.2 Plus Glycohemoglobin Analyzer (Tosoh Medics, Inc., San Francisco, CA). All patients were classified into four groups (Q1, Q2, Q3, and Q4) by quartile of SHR, with Q1 as the reference group.

### Assessment of mortality

The mortality data were downloaded from the National Death Index (NDI) death certificate records provided by NCHS, and the mortality data were updated to December 31, 2019. The study endpoints were all-cause mortality and cardiovascular mortality. The reasons for death were determined by the International Statistical Classification of Diseases, 10th Revision (ICD-10). All-cause mortality was defined as death from any cause, including disease of heart (054–068), malignant neoplasms (019–043), accidents (unintentional injuries, 112–123), cerebrovascular diseases (070), DM (046), and other causes. During the follow-up, cardiovascular mortality was defined as death due to heart disease. The follow-up time was calculated from the baseline interview to the date of death or December 31, 2019.

### Statistical analysis

R software (version 4.3.2) was used to perform the statistical analyses. As required to analyse the NHANES data, sample weights, clustering, and stratification were incorporated into all analyses [16]. The baseline characteristics are shown by quartile of SHR. Continuous variables are presented as mean ± standard deviation (SD). Categorical variables are shown as frequency counts and percentages. P values were calculated by one-way ANOVA for continuous variables and by Pearson’s chi-square test for categorical variables. The incidences of all-cause mortality and cardiovascular mortality were calculated during the follow-up. The log-rank test and Kaplan-Meier (K-M) survival analyses were performed to explore differences in event-free survival between the four groups. A multivariate Cox proportional hazards regression model was applied to calculate the prognostic value of SHR. Three Cox regression models adjusting for different confounding factors were built. Model 1 was not adjusted for any covariates. Model 2 was adjusted for age, sex, and race. Model 3 was adjusted for age, sex, race, BMI, smoking status, alcohol use, hypertension, CHD, CKD, anemia, and TG. We used multiple imputation for missing values. To explore the association between SHR and mortality, Cox proportional hazards regression models with restricted cubic spline (RCS) analyses were performed with four knots. In the RCS model, we also adjusted for confounding factors: age, sex, race, BMI, smoking status, alcohol use, hypertension, CHD, CKD, anemia, and TG. If the relationship was nonlinear, we estimated the threshold value and selected the inflection point with the highest likelihood. We used a two-piecewise Cox proportional hazards model on both sides of the inflection point to investigate the association between SHR and the risk of mortality. Subgroup analyses were also performed to assess the influence of SHR on all-cause mortality and cardiovascular mortality in different subgroups stratified by age (< 60 years, and ≥ 60 years), sex, race (Mexican American, other Hispanic, non-Hispanic white, non-Hispanic black, other races), BMI (underweight, normal, overweight, obese), and diabetes status (diabetes, prediabetes). A *P* value < 0.05 was defined as statistically significant.

## Results

### Baseline characteristics

A total of 11,160 patients with diabetes or prediabetes were included. Their baseline characteristics stratified by quartile of SHR are summarized in Table 1. The average age of the included patients was 57.4 years, and 55.2% of the patients were female. The mean SHR in the Q1, Q2, Q3, and Q4 quartiles was 0.78, 0.91, 0.99, and 1.15, respectively. Patients with a lower SHR were more likely to be older, female, non-Hispanic black, and never smokers than patients in the highest quartile. Among participants in Q1 group, 1622 (47.8%) had consumed 1–5 drinks per month, and 1248 (36.8%) were nondrinkers. Importantly, significant differences were observed between the different quartiles regarding laboratory data, patients in the highest quartiles having significantly lower total cholesterol, HDL-C and LDL-C.

**Table 1:** Baseline demographic and clinical data of four groups

	Q1 (≤ 0.85)	Q2 (0.85–0.93)	Q3 (0.93–1.02)	Q4 (≥1.02)	Total	*P*
SHR, mean (SD)	0.78 (0.08)	0.91 (0.02)	0.99 (0.02)	1.15 (0.16)	0.94 (0.16)	< 0.001
Age, years, mean (SD)	57.4 (15.6)	55.5 (16.1)	52.8 (16.9)	53.5 (17.4)	55.0 (16.5)	< 0.001
Female, n (%)	1872 (55.2)	1320 (48.4)	1060 (43.3)	980 (37.8)	5232 (46.9)	< 0.001
BMI, kg/m^2^, n (%)						0.024
underweight (< 18.5)	32 (0.9)	24 (0.9)	28 (1.1)	25 (1.0)	109 (1.0)	
normal (18.5–25)	756 (22.3)	514 (18.9)	505 (20.6)	527 (20.3)	2304 (20.6)	
overweight (25–30)	1081 (31.9)	926 (34.0)	864 (35.3)	890 (34.3)	3761 (33.7)	
obese (≥ 30)	1522 (44.9)	1262 (46.3)	1050 (42.9)	1152 (44.4)	4986 (44.7)	
Race, n (%)						< 0.001
Mexican American	370 (10.9)	322 (12.2)	330 (13.5)	342 (13.2)	1374 (12.3)	
Other Hispanic	379 (1.2)	312 (11.4)	273 (11.2)	291 (11.2)	1255 (11.2)	
Non-Hispanic White	1073 (31.6)	1110 (40.7)	1082 (44.2)	1143 (44.1)	4408 (39.5)	
Non-Hispanic Black	1069 (31.5)	528 (19.4)	402 (16.4)	482 (18.6)	2481 (22.2)	
Other races	502 (14.8)	444 (16.3)	360 (14.7)	336 (13.0)	1641 (14.7)	
Alcohol use, n (%)						< 0.001
Nondrinker	1248 (36.8)	859 (31.5)	657 (48.3)	693 (26.7)	3457 (31.0)	
1–5 drinks/month	1622 (47.8)	1339 (49.1)	1187 (48.5)	1220 (47.0)	5368 (49.1)	
5–10 drinks/month	191 (5.6)	170 (6.2)	211 (8.6)	212 (8.2)	784 (7.0)	
≥ 10 drinks/month	332 (9.8)	358 (13.1)	392 (16.0)	469 (18.1)	1551 (13.9)	
Smoking status, n (%)						< 0.001
Never	1797 (53.0)	1453 (53.4)	1253 (51.2)	1267 (48.9)	5770 (51.7)	
Former	862 (25.4)	746 (27.4)	705 (28.8)	823 (31.8)	3136 (28.1)	
Current	734 (21.6)	522 (19.2)	487 (19.9)	502 (19.4)	2245 (20.1)	
Hypertension, n (%)	2027 (59.7)	1445 (53.0)	1269 (51.9)	1495 (57.6)	6236 (55.9)	< 0.001
Coronary heart disease, n (%)	277 (8.2)	198 (7.3)	163 (6.7)	207 (8.0)	845 (7.6)	0.147
Congestive heart failure, n (%)	190 (5.6)	111 (4.1)	94 (3.8)	125 (4.8)	520 (4.7)	< 0.001
Chronic kidney disease, n (%)	165 (4.9)	100 (3.7)	93 (3.8)	136 (5.2)	494 (4.4)	0.009
Anemia, n (%)	197 (5.8)	72 (2.6)	53 (2.2)	98 (3.8)	419 (3.8)	< 0.001
TC, mmol/L, mean (SD)	5.0 (1.1)	5.2 (1.1)	5.0 (1.0)	5.0 (1.1)	5.0 (1.1)	< 0.001
LDL-C, mmol/L, mean (SD)	3.0 (1.0)	3.1 (1.0)	3.0 (0.9)	2.9 (0.9)	3.0 (1.0)	< 0.001
HDL-C, mmol/L, mean (SD)	1.4 (0.4)	1.3 (0.4)	1.3 (0.4)	1.3 (0.4)	1.3 (0.4)	< 0.001
TG, mmol/L, mean (SD)	1.4 (1.0)	1.5 (1.2)	1.6 (1.4)	1.8 (1.8)	1.6 (1.4)	< 0.001
HbA1c, %, mean (SD)	6.5 (1.2)	6.0 (0.9)	5.9 (1.1)	6.1 (1.7)	6.1 (1.3)	< 0.001
FPG, mmol/L, mean (SD)	5.8 (1.3)	6.3 (1.3)	6.6 (1.8)	8.3 (3.6)	6.8 (2.3)	< 0.001
HGB, mg/dl, mean (SD)	13.7 (1.6)	14.2 (1.5)	14.4 (1.5)	14.5 (1.6)	14.2 (1.6)	< 0.001
Diabetes, n (%)	1002 (29.5)	2197 (80.6)	1878 (76.7)	1357 (52.4)	7824 (70.1)	< 0.001
Prediabetes, n (%)	2391 (70.5)	529 (19.4)	569 (23.3)	1236 (47.6)	3336 (29.9)	< 0.001

P values were calculated using either Student’s t-test or the chi-square test

BMI body mass index, FPG fasting plasma glucose, HbA1c glycosylated hemoglobin type A1c, HDL-C high-density lipoprotein cholesterol, LDL-C low-density lipoprotein cholesterol, TC total cholesterol, TG triglyceride, SD standard deviation.

### Clinical outcomes for all-cause and cardiovascular mortality

**Fig. 2:**
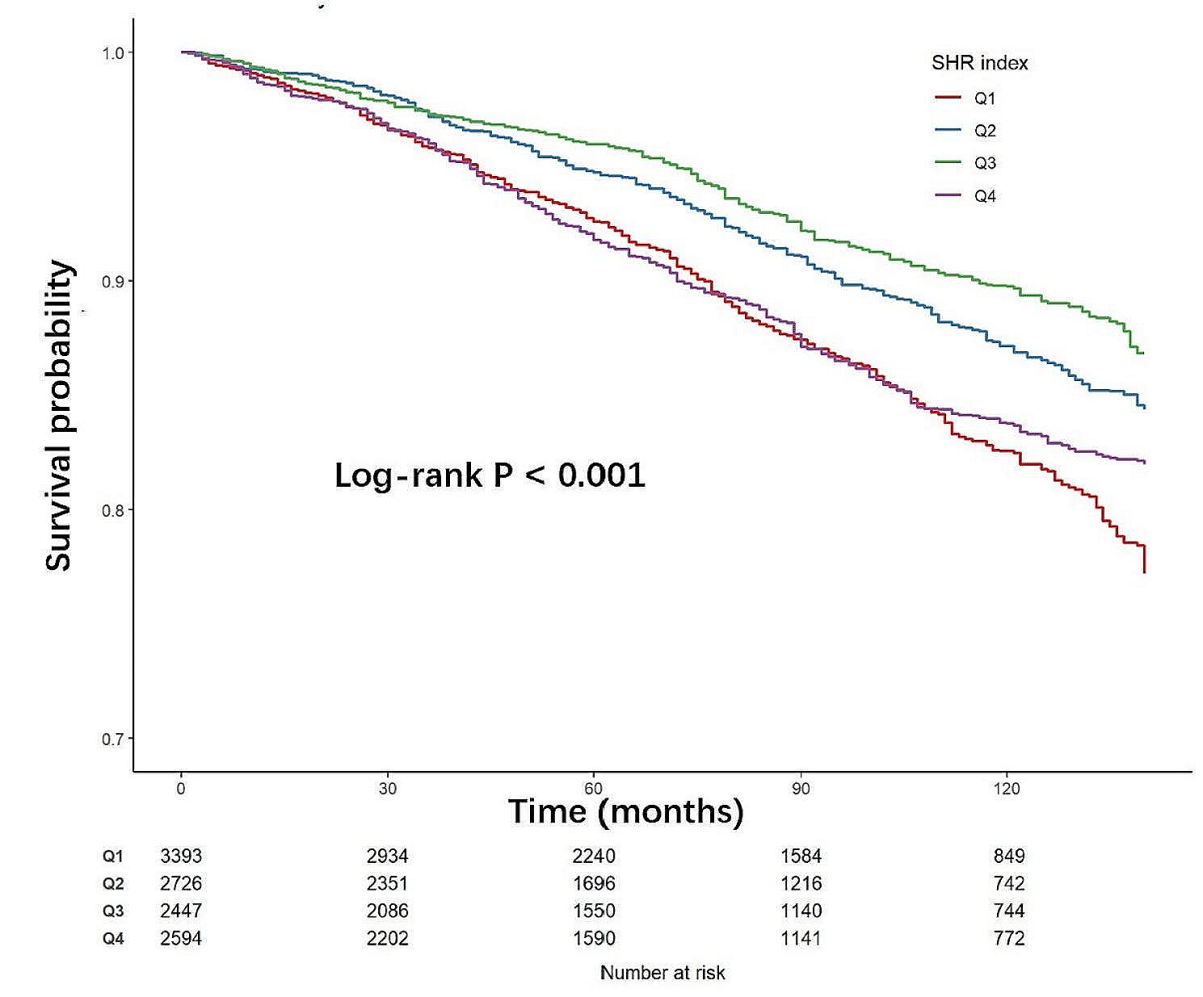
K-M analyses for all-cause mortality among the four groups. Q1–Q4 quartiles 1–4, SHR stress hyperglycemia ratio

During a mean follow-up period of 84.9 months, a total of 1538 all-cause deaths were recorded, for an all-cause mortality rate of 1061/100,000 person-years, while 410 cardiovascular deaths were recorded, for a cardiovascular mortality rate of 33/100,000 person-years. K-M survival analyses showed a significant difference in the incidence of all-cause mortality between the four groups during follow-up, the lowest all-cause mortality being in quartile 3 (log-rank *P* < 0.001). The details of the of K-M survival analyses are presented in Fig. 2. Table 2 shows the three Cox regression models used to evaluate the correlation between SHR and all-cause mortality. Without adjusting for any covariates, the hazard ratios (HRs) and confidence intervals (CIs) from the first quartile to the fourth quartile were 1.00 (reference), 0.70 (0.57, 0.85), 0.60 (0.52–0.70), and 0.89 (0.74–10.7), respectively. In model 2, age, sex, and race were adjusted, and the HRs and 95% CIs were 1.00 (reference), 0.80 (0.67–0.96), 0.82 (0.71–0.96), and 1.24 (1.03–1.49), respectively (all *P* values < 0.05). In addition, in model 3, after adjusting for age, sex, race, BMI, smoking status, alcohol use, hypertension, CHD status, CKD status, anemia, and TG, the HRs and 95% CIs were 1.00 (reference), 0.83 (0.70–0.99), 0.85 (0.74–0.99), and 1.30 (1.08–1.57), respectively. Table 2 also shows the Cox regression models for cardiovascular mortality. Without adjusting for covariates, the HRs and 95% CIs were 1.00 (reference), 0.74 (0.50–1.08), 0.47 (0.32–0.69), and 0.67 (0.50–0.91). Model 2 was adjusted for age, sex, and race, and the HRs and 95% CIs were 1.00 (reference), 0.86 (0.60–1.23), 0.66 (0.45–0.96), and 0.94 (0.71–1.25). In model 3, we further adjusted for BMI, smoking status, alcohol use, hypertension, CHD, CKD, anemia, and TG beyond model 2. The HRs and 95% CIs were 1.00 (reference), 0.88 (0.62–1.25), 0.68 (0.46–0.99), and 0.95 (0.72–1.25).

**Table 2:** Cox regression models for the association between the SHR and mortality

	Quantiles of the SHR				
	Q1	Q2	Q3	Q4	*P* for trend
**All-cause mortality**					
Number of deaths	510	332	280	416	
Model 1 h (95% CI) *P*-value	1	0.70 (0.57, 0.85) < 0.001	0.60 (0.52, 0.70) < 0.001	0.89 (0.74, 10.7) 0.226	< 0.001
Model 2 h (95% CI) *P*-value	1	0.80 (0.67, 0.96) 0.017	0.82 (0.71, 0.96) 0.012	1.24 (1.03, 1.49) 0.026	< 0.001
Model 3 h (95% CI) *P*-value	1	0.83 (0.70, 0.99) 0.037	0.85 (0.74, 0.99) 0.038	1.30 (1.08, 1.57) 0.006	< 0.001
**CVD mortality**					
Number of deaths	150	93	69	98	
Model 1 h (95% CI) *P*-value	1	0.74 (0.50, 1.08) 0.117	0.47 (0.32, 0.69) < 0.001	0.67 (0.50, 0.91) 0.009	< 0.001
Model 2 h (95% CI) *P*-value	1	0.86 (0.60, 1.23) 0.406	0.66 (0.45, 0.96) 0.032	0.94 (0.71, 1.25) 0.665	0.052
Model 3 h (95% CI) *P*-value	1	0.88 (0.62, 1.25) 0.489	0.68 (0.46, 0.99) 0.049	0.95 (0.72, 1.25) 0.693	0.096

BMI body mass index; CI confidence interval; HR hazard ratio; TG triglyceride; SHR stress hyperglycemia ratio.

Model 1: no covariates were adjusted for

Model 2: Adjusted for age, sex and race

Model 3: Adjusted for age, sex, race, BMI, smoking status, alcohol use, hypertension, coronary heart disease, chronic kidney disease, anemia, and TG

### Non-linear relationships between the SHR and mortality

**Fig. 3:**
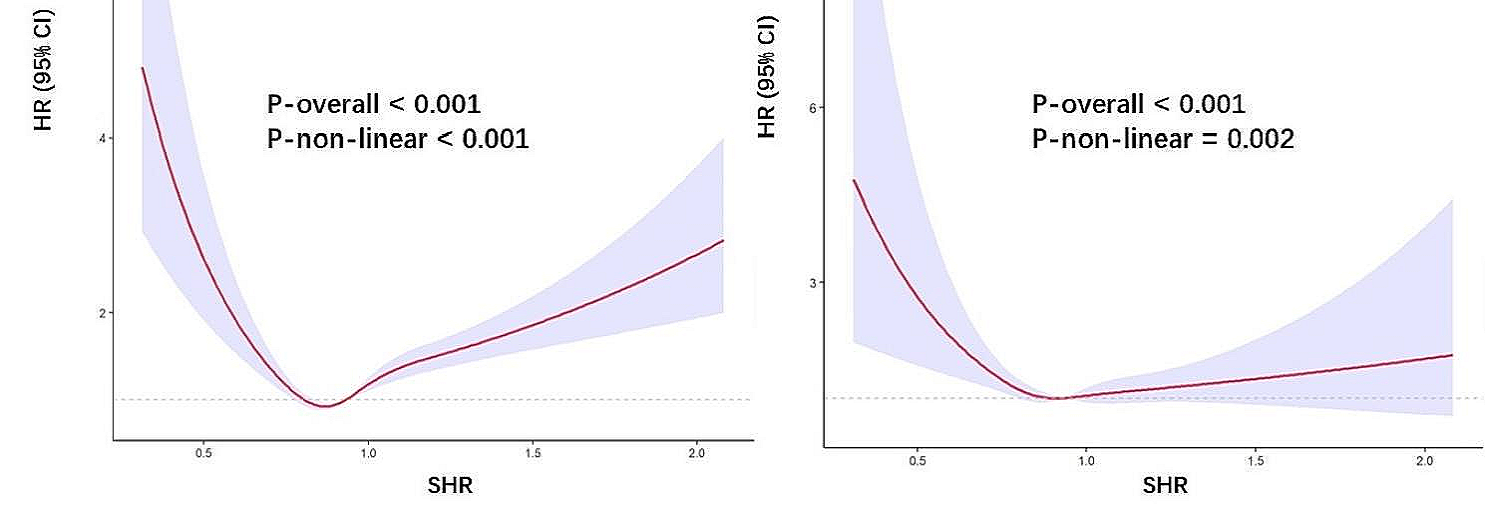
Association between the SHR and all-cause (**A**) and cardiovascular mortality (**B**) in patients with diabetes or prediabetes. Adjusted for age, sex, race, BMI, smoking status, alcohol use, hypertension, coronary heart disease, chronic kidney disease, anemia, and TG. The solid line and purple area represent the estimated values and their corresponding 95% CIs, respectively. SHR stress hyperglycemia ratio; BMI body mass index; CI confidence interval; HR hazard ratio, TG triglyceride

**Fig. 4:**
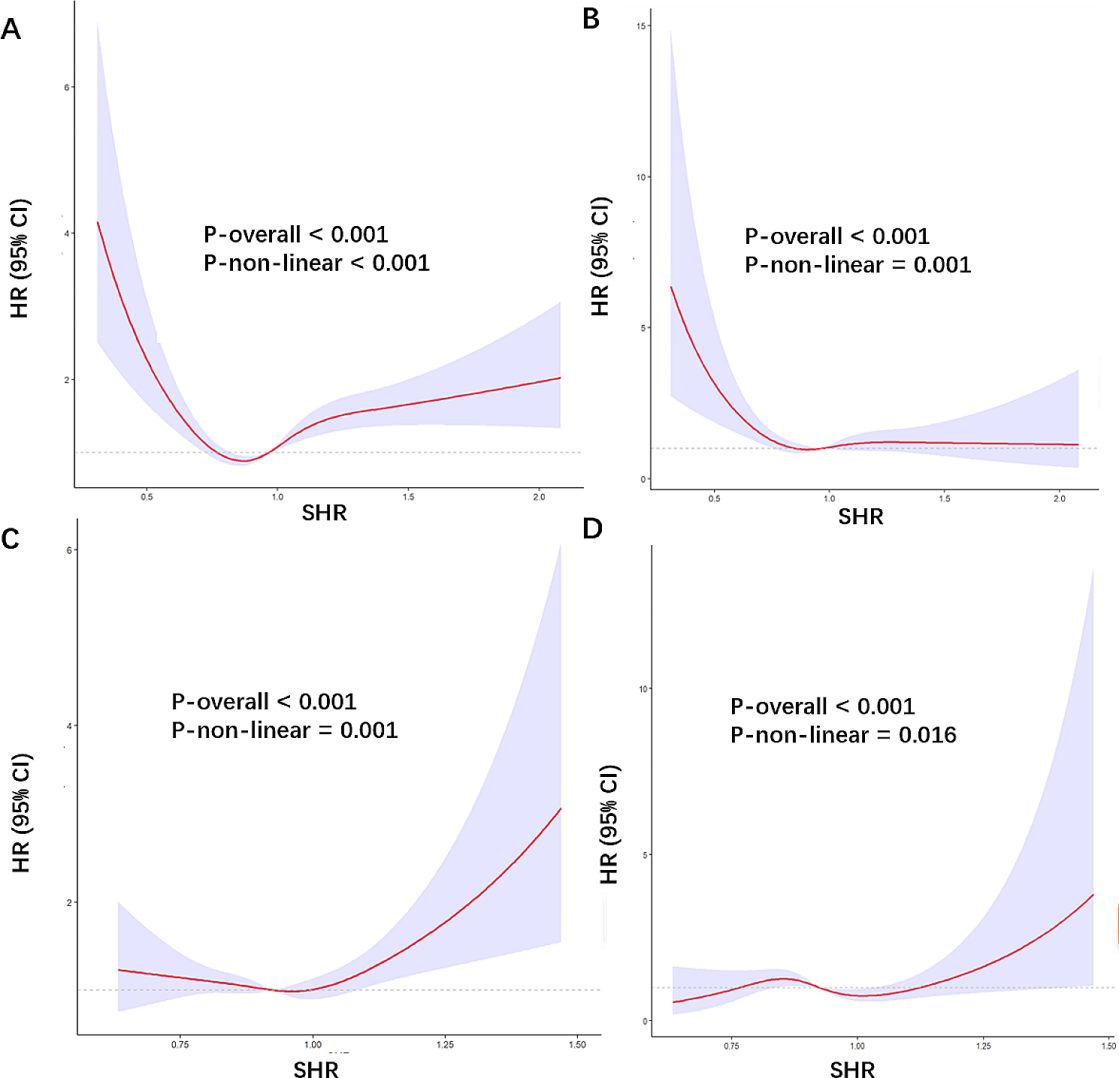
Association between SHR and all-cause (**A**) and cardiovascular mortality (**B**) in patients with diabetes. Association between SHR and all-cause (**C**) and cardiovascular mortality (**D**) in patients with pre-diabetes. Adjusted for age, sex, race, BMI, smoking status, alcohol use, hypertension, coronary heart disease, chronic kidney disease, anemia, and TG. The solid line and purple area represent the estimated values and their corresponding 95% CIs, respectively. SHR stress hyperglycemia ratio; BMI body mass index; CI confidence interval; HR hazard ratio, TG triglyceride

Cox proportional hazards regression models with RCS were used to evaluate the non-linear correlation between the SHR and mortality in patients with diabetes or prediabetes. RCS analysis indicated that there was a U-shaped association between SHR and all-cause mortality (Fig. 3A) even after adjustment for other confounding factors (all *P* values for nonlinearity < 0.05; Fig. 3). The adjusted confounding factors included age, sex, race, BMI, smoking status, alcohol use, hypertension, CHD, CKD, anemia, and TG. There was an L-shaped association between SHR and cardiovascular death (P for nonlinearity < 0.05); when SHR was < 0.93, the HR changed sharply, while it increased slowly when SHR was > 0.93, (Fig. 3B). The value of SHR corresponding to the lowest risk of all-cause mortality according to multivariate-adjusted RCS analyses was 0.87 for the population (Fig. 3**)**. We fitted the association between SHR and mortality using a standard Cox proportional hazards regression model and a two-piecewise Cox proportional hazards regression model. Through these models, we identified the inflection points for all- cause and cardiovascular mortality as 0.87 and 0.93, respectively (Table 3). After adjusting for age, sex, race, BMI, smoking status, alcohol use, hypertension, CHD status, CKD status, anemia, and TG, the risk of all-cause mortality and cardiovascular mortality decreased by 91% (HR 0.09, 95% CI: 0.02–0.33) and 92% (HR 0.08, 95% CI: 0.01–0.44), respectively, as the SHR increased to the inflection points. The risk of all-cause mortality increased as SHR increased when SHR was > 0.87 (HR 2.80, 95% CI: 1.97–3.98). We next studied the diabetes and prediabetes populations separately. In patients with diabetes, there were still a U-shaped and L-shaped associations between the SHR and all-cause mortality and cardiovascular mortality, respectively (Fig. 4A and B). The SHR and all-cause mortality in patients with prediabetes were nearly U-shaped (Fig. 4C), while there was roughly J-shaped association with cardiovascular mortality (Fig. 4D).

**Table 3:** Threshold effect analysis of the SHR on all-cause and cardiovascular mortality in patients with diabetes or prediabetes

	Adjusted HR (95%CI)	*P*-value
**All-cause mortality**		
Total	1.77 (1.36, 2.31)	< 0.001
Fitting by two-piecewise Cox proportional risk model		
Inflection point	0.87	
SHR < 0.87	0.09 (0.02, 0.33)	< 0.001
SHR ≥ 0.87	2.80 (1.97, 3.98)	< 0.001
**Cardiovascular mortality**		
Total	1.60 (0.80, 3.20)	0.182
Fitting by two-piecewise Cox proportional risk model		
Inflection point	0.93	
SHR < 0.93	0.08 (0.01, 0.44)	< 0.001
SHR ≥ 0.93	1.67 (0.84, 3.31)	0.141

The model was adjusted for age, sex, race, BMI, smoking status, alcohol use, hypertension, coronary heart disease, chronic kidney disease, anemia, and TG

BMI body mass index; CI confidence interval; HR hazard ratio; TG triglyceride; SHR stress hyperglycemia ratio.

### Subgroup analysis

Subgroup analyses were performed to evaluate the associations of SHR with mortality in different populations according to age (< 60 years, ≥ 60 years), sex (female, male), race (Mexican American, other Hispanic, non-Hispanic white, non-Hispanic black, other races), BMI (underweight, normal, overweight, obese), and diabetes status (diabetes, prediabetes). The relationship between SHR and mortality among patients with diabetes or prediabetes was consistent across the various subgroups, as depicted in Tables 4 and 5. There was no significant interaction effect between SHR and stratified variables.

**Table 4:** Subgroup analyses of the association between the SHR and all-cause mortality

	All-cause mortality			
	HR 95% (CI)		*P* value	
**SHR**	< 0.87	≥ 0.87		*P* interaction
**Overall**				
**Age, years**				0.587
< 60	Reference	0.92 (0.64, 1.32)	0.631	
≥60	Reference	0.90 (0.78, 1.04)	0.140	
**Sex**				0.052
Female	Reference	1.15 (0.95, 1.38)	0.150	
Male	Reference	0.88 (0.73, 1.06)	0.182	
**BMI**				0.145
underweight (< 18.5)	Reference	1.23 (0.20, 7.54)	0.824	
normal (18.5–25)	Reference	1.29 (1.03, 1.60)	0.027	
overweight (25–30)	Reference	1.04 (0.91, 1.33)	0.772	
obese (≥ 30)	Reference	0.84 (0.65, 1.07)	0.154	
**Race**				0.820
Mexican American	Reference	1.24 (0.79, 1.94)	0.344	
Other Hispanic	Reference	1.08 (0.64, 1.59)	0.680	
Non-Hispanic white	Reference	0.98 (0.82, 1.19)	0.864	
Non-Hispanic black	Reference	1.15 (0.88, 1.50)	0.318	
Other races	Reference	1.00 (0.66, 1.52)	0.996	
**Diabetes status**				0.707
Diabetes	Reference	0.98 (0.80, 1.19)	0.833	
Prediabetes	Reference	0.97 (0.92, 1.15)	0.742	

**Table 5:** Subgroup analyses of the association between the SHR and cardiovascular mortality

	Cardiovascular mortality			
	HR 95%(CI)		*P* value	
**SHR**	< 0.91	≥ 0.91		*P* interaction
**Overall**				
**Age, years**				0.091
< 60	Reference	1.28 (0.71, 2.30)	0.420	
≥60	Reference	0.72 (0.53, 0.97)	0.029	
**Sex**				0.622
Female	Reference	0.95 (0.68, 1.34)	0.777	
Male	Reference	0.82 (0.58, 1.15)	0.248	
**BMI**				0.279
underweight (< 18.5)	Reference	0.48 (0.07, 3.72)	0.453	
normal (18.5–25)	Reference	1.24 (0.70, 2.22)	0.462	
overweight (25–30)	Reference	0.76 (0.48, 1.21)	0.242	
obese (≥ 30)	Reference	0.79 (0.55, 1.13)	0.200	
**Race**				0.286
Mexican American	Reference	1.74 (0.65, 4.71)	0.272	
Other Hispanic	Reference	1.10 (0.42, 2.86)	0.846	
Non-Hispanic white	Reference	0.83 (0.58, 1.19)	0.315	
Non-Hispanic black	Reference	0.79 (0.48, 1.29)	0.348	
Other races	Reference	1.08 (0.42, 2.77)	0.878	
**Diabetes condition**				0.510
Diabetes	Reference	0.75 (0.51, 1.11)	0.149	
Prediabetes	Reference	0.93 (0.64, 1.34)	0.700	

## Discussion

To the best of our knowledge, this is the first study to evaluate the association between SHR and all-cause and cardiovascular mortality in patients with diabetes or prediabetes. The major findings of the current study were as follows: (1) SHR was independently associated with all-cause and cardiovascular mortality in patients with diabetes or prediabetes even after adjusting for potential confounding variables; (2) the association was U-shaped for all-cause mortality, the HRs significantly increasing when SHR was > 0.87; and (3) the association was L-shaped for cardiovascular mortality.

Stress-induced hyperglycemia is common in patients suffering critical illness and leads to insulin resistance (IR), inflammatory reactions, and severe dysfunction of glucose metabolism [17]. The admission blood glucose level cannot reflect the overall hyperglycemia status, as this does not evaluate the chronic glucose level. SHR is a simple and convenient measure for evaluating stress-induced hyperglycemia [11]. SHR has been independently associated with cerebral edema after acute cerebral infarction [6], risk of pulmonary infection during hospitalization [18], severity of coronary artery disease [19], and thrombus burden [20]. In addition to its strong correlation with various diseases, SHR has been used to predict clinical outcomes. In patients with acute myocardial infarction, elevated SHR is significantly associated with long-term all-cause mortality in both American and Chinese cohorts [21]. In patients with acute decompensated heart failure, there was a U-shaped association between SHR and mortality and rehospitalization rates [7]. SHR is also independently associated with the risk of major cardiovascular adverse events (MACE) [22]. The above evidences show that SHR could become a clinical indicator of prognosis.

The current study may be the first to focus on the diabetic or prediabetic populations and to find U-shaped or L-shaped association between SHR and mortality. We included 11,160 diabetic or prediabetic patients in the United States diagnosed from 2005 to 2018, and the median follow-up period was 84.9 months. The results showed that there was a U-shaped relationship between SHR and all-cause mortality and an L-shaped relationship between SHR and cardiovascular mortality. The HR for all-cause mortality increased significantly when the SHR was greater than 0.87 after adjusting for the potential confounding factors. A U-shaped association between SHR and poor prognosis has been seen in previous studies. Yang et al. [8] reported a U-shaped correlation between SHR and the MACE rate and a J-shaped correlation between SHR and in-hospital cardiac death in patients with acute coronary syndrome. Roberts et al. [11] reported a roughly J-shaped association between SHR and critical illness, which is in accordance with the results of our study to some extent. Karakasis et al. conducted a meta-analysis that demonstrated that higher SHR was associated with a significantly greater risk of MACCE and short-term and long-term mortality, which is in accordance with the outcomes of our study to some degree. Their conclusions were consistent between patients with and without diabetes [14]. The mechanisms underlying the U-shaped or L-shaped association remain uncertain, but they may encompass the following aspects.

First, a high SHR is indicative of stress hyperglycemia during both the acute and chronic periods and is associated with adverse outcomes. The mechanism by which a high SHR leads to increased mortality may be explained as follows: (1) Stress hyperglycemia can increase IR and the release of catecholamines, cortisol, glucagon and growth hormones [23–25]; catecholamines can inhibit insulin secretion, modulate glucose transport molecules leading to peripheral IR, and stimulate hepatic gluconeogenesis [26]; (2) hyperglycemia can prompt inflammation, endothelial dysfunction and oxidative stress [27–29]: the rapid increase in glucose causes the overproduction of reactive oxygen species (ROS), and also inflammatory conditions can cause numerous diabatic complications, including microvascular and macrovascular complications [29]. Monnier et al. [27] estimated oxidative stress using the 24-hour urinary excretion rate of free 8-iso prostaglandin F_2α_ (8-iso PGF_2α_) and found that 8-iso PGF_2α_ excretion rates were higher in patients with diabetes. These authors demonstrated that stress hyperglycemia triggers oxidative stress; (3) A high SHR could impair fibrinolysis: which is related to the breakdown of thrombosis. In patients with poorly controlled diabetes, extraordinarily high concentrations of plasminogen activator inhibitor-1 (PAI-1), which indicates hypofibrinolysis, were detected. After lowering the blood glucose, the PAI-1 concentration decreases, which suggests that the glucose level is related to fibrinolysis [30, 31]; (4) Endothelial dysfunction: in patients with high SHR, the balance between dilating factors (e.g. nitric oxide (NO), prostacyclin (PGI_2_)) and constricting factors (endothelin, angiotensin II, TX) shifts towards constriction [32]; (5) Platelet activation: Disturbances in endothelial function and coagulation may activate the initial process of platelet activation and adhesion and subsequent platelet aggregation [33].;(6) Uncontrolled glucose levels may cause harm, including deleterious effects on wound healing, increased risk of infection and prolonged hospitalization, which can lead to noncardiovascular death [34].

Previous studies have reported a J-shaped association between SHR and poor short-term and long-terms prognoses in different populations [8, 11]. Our study is the first to show an L-shaped association which emphasizes the dangerous impact of a low SHR on cardiovascular mortality. A low SHR is associated with a worse long-term prognosis in patients with diabetes or prediabetes. Patients with a very low SHR may experience more hypoglycemic episodes or chronic hyperglycemia (high HbA1c) with current good or excessive glycemic control (low admission glucose). Both situations could be harmful. As mentioned before, chronic hyperglycemia may contribute to adverse outcomes through oxidative stress, inflammation, or IR. The relationship between hypoglycemia and adverse clinical outcomes has been supported by previous studies [35–40]. Zoungas et al. [35] included diabetic patients, 231 of whom had at least one severe hypoglycemic episode. During the follow-up, hypoglycemia was associated with a significant increase in MACE (HR 2.88, 95% CI 2.01–4.12), all-cause mortality (HR 2.69, 95% CI 1.97–3.67), and cardiovascular mortality (HR 1.81, 95% CI 1.19–2.74). Another randomized study found that patients who experienced severe hypoglycemia had a higher incidence of heart failure and kidney disease. They were also more likely to experience MACE, all-cause death, and cardiovascular death than those without hypoglycemia [36]. Our study is consistent with the above studies to some extent. In addition, patients with diabetes or prediabetes are more likely to experience hypoglycemic episodes spontaneously or iatrogenically. This may be attributed to incorrect use of insulin or oral medications, extended periods of fasting, or digestive difficulties. The mechanism by which hypoglycemia increases cardiovascular mortality may involve the following: (1) Hypoglycemia induces platelet hyperactivity through the elevation of inflammatory and oxidative stress markers [41]; the concentration of fatty acids, including 10-nonadecenoate, linolenate and dihomo-linoleate increase; and molecules contributing to cardiovascular complications, such as fatty-acid-binding protein-3, are also altered during hypoglycemia [42]. (2) More cardiac arrhythmias: An in vitro study has shown that cardiac arrhythmias are often triggered by hypoglycemia through effects on cardiac repolarization and changes in cardiac autonomic activity. In patients with type 2 diabetes with cardiovascular risk, a heart rate-corrected QT interval, > 500ms and abnormal T-waves were observed during hypoglycemia [43]; (3) Causing the activation of the sympatho-adrenal system: acute hypoglycemia causes a pronounced physiological response, releases the epinephrine, and provokes hemodynamic changes. The hemodynamic changes lead to the an increase in heart rate, peripheral systolic blood pressure, myocardial contractility, stroke volume, and cardiac output [44]. Transient cardiac stress may have a dangerous impact on older people with diabetes especially individuals with CHD.

In summary, previous studies have demonstrated an association between stress hyperglycemia and clinical outcomes. These findings, in conjunction with our own, highlight the clinical significance of maintaining an optimal SHR, as deviations to either extremely high or low levels can result in detrimental health consequences.

Several limitations of our work should be noted. First, even after we adjusted for several potential confounding factors in the multivariate model, SHR still might have been affected by other factors. Second, this was an observational study based of patients with diabetes or prediabetes in the United States population. Prospectively studies are needed to further test this relationship and the underlying mechanisms involved. Third, the subgroup analyses did not meet the optimal information size criterion, which may have caused bias in the subgroup analyses.

## Conclusions

In the present study, the index SHR was found to be a valuable index for predicting the risk of all-cause and cardiovascular mortality in patients with diabetes or prediabetes. There was a U-shaped association between SHR and all-cause mortality and an L-shaped associations between SHR and cardiovascular mortality, in which the inflection points of SHR for poor prognosis were 0.87 and 0.93, respectively. Large-scale, multicenter prospective studies should be performed to assess the predictive value of SHR and to investigate the underlying mechanisms of the U-shaped and L-shaped associations.

## Data Availability

The datasets presented in this article are not readily available because research data is confidential. Requests to access the datasets should be directed to doctortangmin@yeah.net.
